# BODIPY Dyes as Probes and Sensors to Study Amyloid-β-Related Processes

**DOI:** 10.3390/bios10120192

**Published:** 2020-11-27

**Authors:** Sergei V. Dzyuba

**Affiliations:** Department of Chemistry and Biochemistry, Texas Christian University, Fort Worth, TX 76129, USA; s.dzyuba@tcu.edu

**Keywords:** amyloids, protein folding, Alzheimer’s disease, Aβ1-42, BODIPY, fluorescent dyes, environment-sensitive probes

## Abstract

Amyloid formation plays a major role in a number of neurodegenerative diseases, including Alzheimer’s disease. Amyloid-β peptides (Aβ) are one of the primary markers associated with this pathology. Aβ aggregates exhibit a diverse range of morphologies with distinct pathological activities. Recognition of the Aβ aggregates by using small molecule-based probes and sensors should not only enhance understanding of the underlying mechanisms of amyloid formation, but also facilitate the development of therapeutic strategies to interfere with amyloid neurotoxicity. BODIPY (boron dipyrrin) dyes are among the most versatile small molecule fluorophores. BODIPY scaffolds could be functionalized to tune their photophysical properties to the desired ranges as well as to adapt these dyes to various types of conditions and environments. Thus, BODIPY dyes could be viewed as unique platforms for the design of probes and sensors that are capable of detecting and tracking structural changes of various Aβ aggregates. This review summarizes currently available examples of BODIPY dyes that have been used to investigate conformational changes of Aβ peptides, self-assembly processes of Aβ, as well as Aβ interactions with various molecules.

## 1. Introduction

Protein folding is one of the most complex, challenging, and fascinating phenomena of modern science and medicine. Amyloids are a group of peptides and proteins that are prone to misfolding, i.e., adaptation of non-native conformations, assemblies, etc. In general, biomolecule misfolding or abnormal interactions lead to formation of various types of deposits, which are typically pathological in nature [1,2]. Although specific amino acid sequences are typically required for amyloid formation, abundant globular proteins could also be forced into amyloid-like structures [3,4].

**Figure 1:**
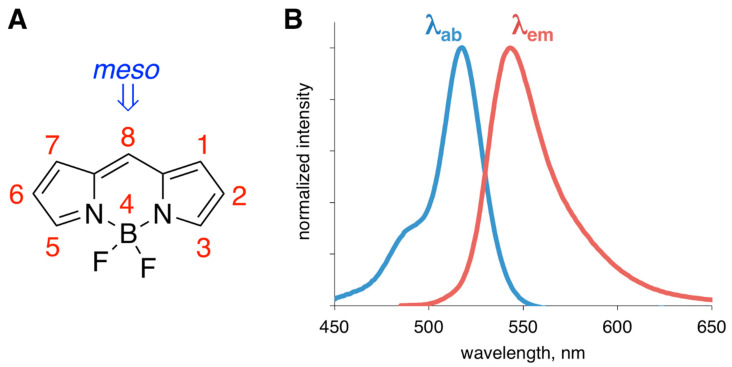
(**A**) Structure of BODIPY core and the numbering system; position-8 is also referred to as *meso*-position. (**B**) Representative absorption (blue, β_ab_ is the absorption maximum) and emission (red, β_em_ is the emission maximum) spectra of a generic BODIPY dye.

In order to track diverse range of intermediates leading to amyloid formation, various types of probes and sensors are required. Notably, due to synthetic accessibility of broad range of functionalities, small molecular probes and sensors (dyes) have been widely used to monitor various biological processes, including those related to amyloids. Among many useful fluorescent dyes, BODIPY (boron dipyrrin or 4,4-difluoro-4-bora-3a,4a-diaza-s-indacene) dyes (Figure 1) have received a large amount of attention due to their structural and functional versatility, which contributes to their use in a very diverse range of applications, in particular as probes and sensors [5,6,7,8]. It should also be pointed that some BODIPY dyes have been mentioned in numerous recent reviews on small molecule probes and methods for amyloid detection [9,10,11,12,13,14,15,16]. However, no detailed, comprehensive analyses and summaries in regard to the BODIPY dyes and amyloid-β peptides have been made to date.

In light of increasing number of accounts, as well as diversity of reported structures and approaches, it is warranted to summarize the information that specifically deals with BODIPY dyes as probes and sensors of conformational changes, self-assembly processes, and interactions of amyloid-β peptides. This review is based on the information available in the primary literature accounts that are published in scientific journals. Patents that are related to amyloids and BODIPY dyes were not included in this review due to scarcity of experimental information typically given in patent literature.

## 2. Amyloid-β and Alzheimer’s Disease

**Figure 2:**
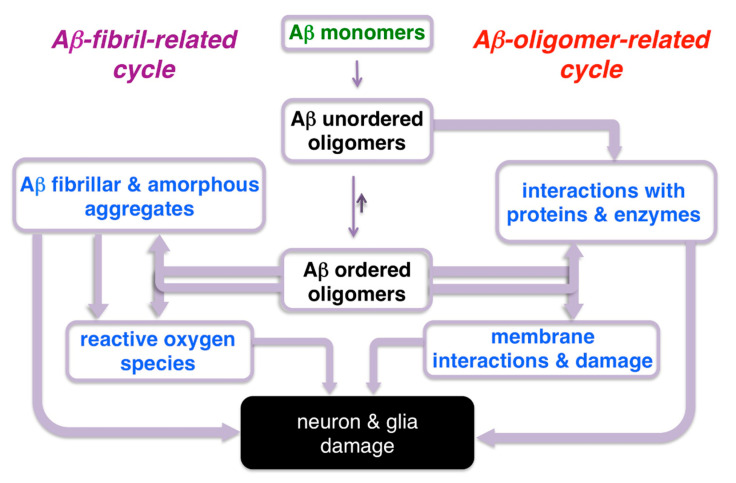
Simplified schematic representation of amyloid-β-related oligomers and fibrillar aggregate interactions that lead to neuronal damage in Alzheimer’s disease (AD).

Amyloid peptides undergo self-assembly processes that lead to formation of a diverse range of structures with various degrees of neurotoxicity. So-called amyloid-β or Aβ peptides have been the cornerstone of the amyloid hypothesis of Alzheimer’s disease (AD) [17,18,19]. Aβ peptides are typically 37–43 amino acid long sequences, with Aβ1-40 being the most prevalent, and Aβ1-42 being the most neurotoxic, i.e., the most prone to formation of oligomeric and higher order aggregates. With regard to Aβ neurotoxicity, Aβ fibril-dependent [20,21,22] and Aβ oligomer-dependent [23,24,25,26,27,28] pathways (or a combination of both) could be envisioned (Figure 2). Although some earlier studies suggested that monomers of amyloid-β might not have deleterious effects [29,30], some recent accounts might implicate the monomeric form’s involvement in some pathological pathways [31].

### General Comments on the Preparation of Aβ Monomers, Oligomers, and Fibrils

Typically, lyophilized peptides are treated either with hexafluoroisopropanol (HFIP), dilute NaOH, or NH_4_OH solution to disaggregate Aβ, sometimes followed by sonication. Subsequently, HFIP is removed, and the resulting film is dissolved in appropriate buffer (with or without addition of organic solvents, such as DMSO, for example); in the case of NaOH (or NH_4_OH) treatments, direct dilution into appropriate buffer is carried out to obtain a desired concentration of the Aβ stock solution. Considering that Aβ1-40 and Aβ1-42 do undergo continuous aggregation, in most aqueous buffers, storage of high concentration (e.g., 1 mM–100 µM) stock should be minimized. Below is a brief outline and some notes on representative preparations of Aβ monomers, oligomers, and fibrils.

- Aβ monomers

In view of high aggregation propensity of Aβ peptides, obtaining an exclusively monomeric form is challenging. It is typically assumed that freshly prepared solutions of Aβ peptides contain mostly monomers. This is often confirmed by antibody binding/dot blot or SDS-PAGE assays. Yet, the presence of higher order aggregates cannot be excluded. The measurements of the diluted solutions of Aβ should be initiated immediately following the preparation of the solutions.

- Aβ oligomers

Stock solution of disaggregated Aβ peptides is diluted to the desired peptide concentration, and the resulting solution is allowed to equilibrate for several hours prior to the measurements. In some cases, additional steps, such as centrifugation or dialysis, could also be introduced [32].

- Aβ fibrils

Typically, allowing the diluted Aβ oligomer solution to equilibrate for up to 5–7 days at room temperature (or 1–2 days at 37 °C), with or without additional perturbations [32], leads to the formation of the Aβ fibrils.

The specific details on the preparation of Aβ aggregates (oligomers and fibrils) that have been used for establishing sensing abilities of BODIPY dyes have been typically based on literature precedents. Without a doubt, from a practical point of view, robust and *reproducible* preparations of various forms of Aβ aggregates are required. More importantly, regardless of the desired form of the amyloid-β, uniform, standard conditions for Aβ preparations (i.e., specific/fixed concentration of Aβ, disaggregation agents, concentration and pH of the buffer, time of incubation, etc.) should be utilized to allow for meaningful comparison among various amyloid probes and sensors.

In addition, it should also be noted that most studies on assessing aggregation propensity (and neurotoxicity) of amyloid peptides are performed using homogeneous, synthetically made Aβ peptides with specific and fixed sequences and lengths. It could be argued that these model studies might not be providing realistic information about the behavior of Aβ in biological settings, where heterogeneous (in regard to composition, i.e., length of peptides) mixtures of Aβ peptides are present [23]. Furthermore, the presence of other cellular components and macromolecular crowding effects should be considered, not only in regard to amyloids themselves, but also in regard to the probes and sensors.

## 3. BODIPY-Based Sensors for Monitoring Aggregation and Conformational Changes of Amyloids

BODIPY dyes are among the most versatile classes of small molecule fluorophores, primarily because their photophysical properties could be fine-tuned over wide ranges using fairly straightforward synthetic protocols [33,34,35,36]. Numerous synthetic approaches, including direct synthesis via condensation of appropriately substituted pyrroles with various carbonyl-containing compounds, with subsequent incorporation of BF_2_– or B-O– functionalities, have been introduced [33,34]. As a complementary approach, so-called postfunctionalization of BODIPY scaffold has also been exploited to expand the scope of functionally and structurally diverse BODIPY dyes [37].

### 3.1. Non-Covalent Interactions of BODIPY Dyes with Aβ Fibrils

**Scheme 1:**
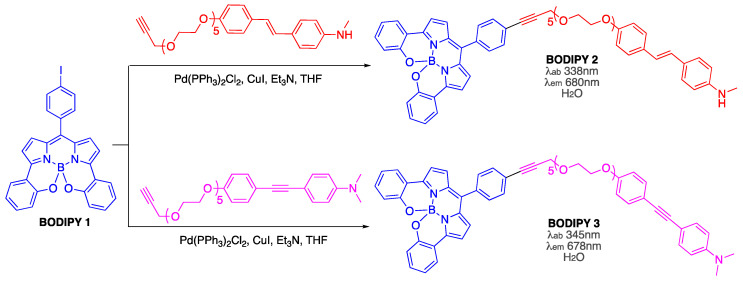
Synthesis of BODIPY probes as suggested stains for amyloid aggregates from AD brain.

The first suggestion that BODIPY dyes could be used for visualizing Aβ fibrillar aggregates [38] was based on the assumption that by conjugating **BODIPY 1** with proven amyloid-binders (e.g., 4-[2-[4-(methylamino)phenyl]ethenyl]phenol and 4-[2-[4-(methylamino)phenyl]ethynyl]phenol and their corresponding glycol-containing derivatives, which exhibited low nM binding affinities towards amyloid-β from AD brain homogenates [39,40]) could yield novel amyloid sensors and stains **BODIPY 2** and **BODIPY 3** (Scheme 1). However, the ability of these probes to interact with Aβ aggregates is yet to be explored.

**Scheme 2:**
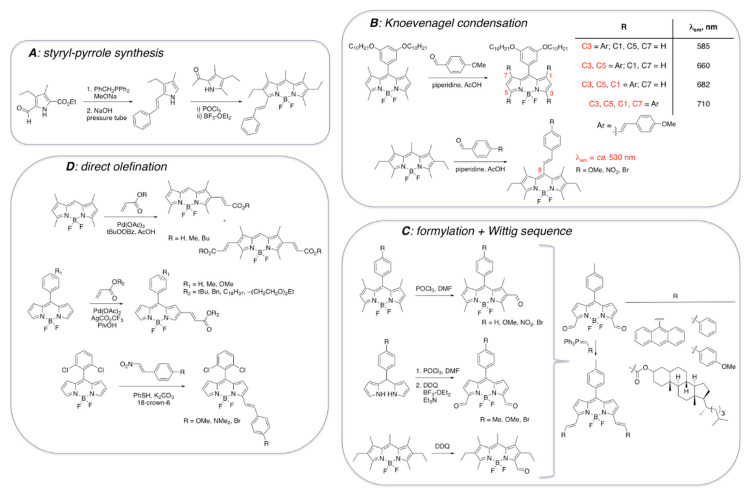
Some general synthetic approaches towards styryl-containing BODIPY dyes. (**A**) BODIPY synthesis using styryl-containing pyrrole; (**B**) introduction of extended conjugation on BODIPY scaffold by using Knoevenagel condensation; (**C**) a two-step approach to BODIPY dyes with extended conjugation; (**D**) access to styryl-BODIPY dyes via direct olefination of BODIPY scaffolds.

It should be noted that various synthetic approaches to incorporate styryl functionalities onto BODIPY core have been reported (Scheme 2) [41,42,43,44,45,46,47,48,49,50]. Although styryl-containing pyrroles (Scheme 2A) could be used for the synthesis of the corresponding BODIPY dyes [41], synthesis of styryl-containing BODIPYs is more conveniently accomplished by using several relatively straightforward and facile postfunctionalization protocols (Scheme 2B–D). Knoevenagel condensation between methyl-containing BODIPY dyes and appropriate aldehydes have been used as the main route to introduce the alkenyl moieties, mostly in view of the synthetic accessibility and/or commercial availability of the starting materials (Scheme 2B) [42,43]. In addition, incorporation of the formyl group in various positions of the BODIPY core was shown to occur under various sets of conditions (Scheme 2C) [44,45,46]. Subsequently, these precursors could be converted to styryl-containing BODIPYs via Wittig reaction [47], for example. Several other protocols have also been established to directly introduce ethenyl functionalities onto BODIPY scaffold (Scheme 2D) [48,49,50]. However, the extended conjugation in position 3 and position 5, as opposed to other positions, is typically preferred due to a more pronounced red shift of the emission maxima for these BODIPY derivatives (Scheme 2B).

**Scheme 3:**
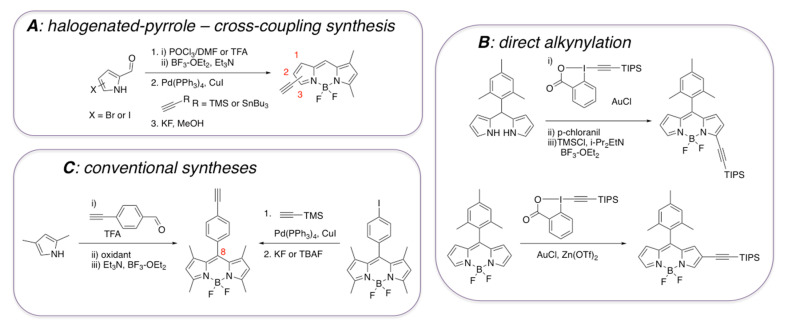
Some synthetic approaches towards ethynyl-containing BODIPY dyes. (**A**) synthesis of ethynyl-containing BODIPYs from halogenated pyrroles; (**B**) introduction of the ethynyl group on either substituted dipyrromethene or BODIPY scaffold via C-H functionalization; (**C**) one-pot synthesis of ethynyl-containing BODIPY or alkynylation of BODIPY scaffold.

Compared to styryl-containing BODIPY dyes, from the structural and functional diversity points of view, ethynyl-containing BODIPY dyes have received less attention (Scheme 3). BODIPY dyes with ethynyl functionality in the 1, 2, or 3-positions are not particularly stable (Scheme 3A) [51,52], and thus they should be either silyl-protected (Scheme 3B) [53] or should be converted to other derivatives [51,52]. On the other hand, the presence of the ethynyl-group in the *meso* position (directly attached to BODIPY or through an aromatic group), gives thermo- and photo-stable dyes, which could be synthesized in a fairly straightforward manner using commercially available starting materials (Scheme 3C) [54,55,56,57]. Considering that some ethynyl- and diyne-containing BODIPY dyes have been used as environment-sensitive probes, primarily reporting on the viscosity fluctuations in various types of media, including biological systems [58,59], it could be argued that some of these BODIPY dyes could also have some potential for detecting amyloids.

The ability of some styryl-containing BODIPY dyes to interact with Aβ aggregates has been explored in several accounts [60,61,62,63]. In general, a viable probe for detecting and staining Aβ aggregates from AD homogenates should fulfill the following criteria: (a) a near-IR emission, preferably above the 650 nm range; (b) specific labeling of the Aβ aggregates, with rapid clearing of the unbound probe; and (c) change of photophysical characteristics upon binding (i.e., emission intensity, wavelength, etc.).

**Scheme 4:**
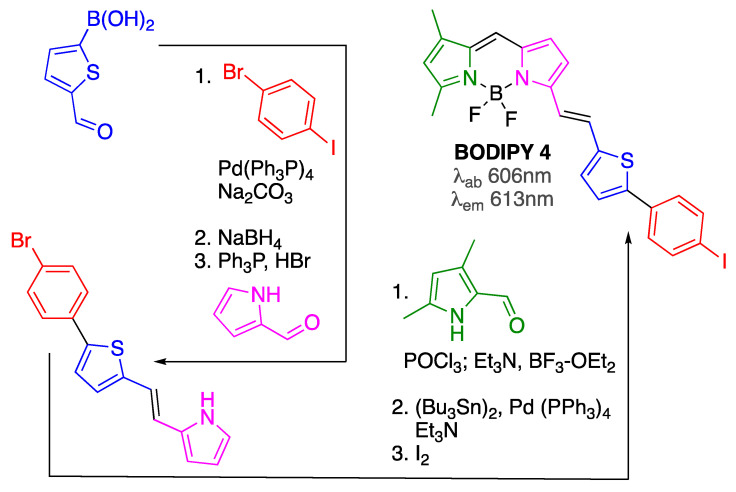
Synthesis of **BODIPY 4**. ^125^I-containing BODIPY could be obtained from the SnBu_3_-BODIPY intermediate upon treatment with Na^125^I.

Initially, **BODIPY 4**, which was prepared following a multi-step procedure from commercially available materials (Scheme 4), was designed to function as both a nuclear (upon incorporation of ^125^I) and fluorescent imaging probe for in vitro (and potentially for in vivo) imaging of amyloid aggregates present in AD brains [60]. **BODIPIY 4** was able to show reasonable in vitro binding affinity (around 100 nM range) towards Aβ1-42 aggregates. This dye showed an emission around 615 nm, thus approaching the characteristics of a viable Aβ imaging probe.

**Scheme 5:**
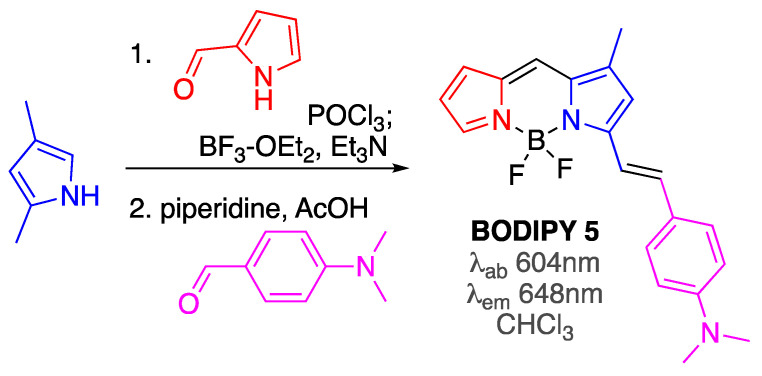
Synthesis of **BODIPY 5**.

Subsequent optimization of the structure revealed that incorporation of the dimethylamino group on the substituent in position 5 could not only produce **BODIPY 5** (Scheme 5) with enhanced in vitro affinity towards Aβ aggregates (K_d_ = 44 nM, i.e., around 2.5 improvement compared to **BODIPY 4**), but also red shifted the emission maximum closer to the desired near-IR range (*λ*_em_ ≈650 nm) [61]. It should also be noted that **BODIPY 5** was synthesized only in two steps from commercially available materials (Scheme 5), which should facilitate the use of this dye in amyloid-related and other applications.

**Figure 3:**
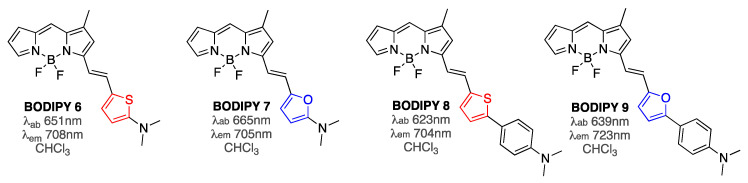
Structures of **BODIPY 6–9**.

Further fine-tuning of the structure of the substituent at position 3 of the BODIPY core, i.e., adjusting the nature of the tether between the BODIPY and the amine functionality (essentially combing the features of **BODIPY 4** and **BODIPY 5**) yielded **BODIPY**s **6**–**9** (Figure 3), which exhibited Aβ-binding affinities in the 20–150 nM range [62]. Emission of these probes was further red shifted to the desired in vivo imaging studies 700 nm range. Remarkably, these probes showed drastically enhanced emission intensities in the presence of equimolar amounts of Aβ1-42 fibrils, with **BODIPY 9** exhibiting the largest increase of 23-fold. Since emission enhancement in the presence of large excess of bovine serum albumin (BSA; 680 µM) was only around twofold for all **BODIPY**s **6**–**9**, these sensors showed very high specificity for amyloid aggregates [62].

**Figure 4:**
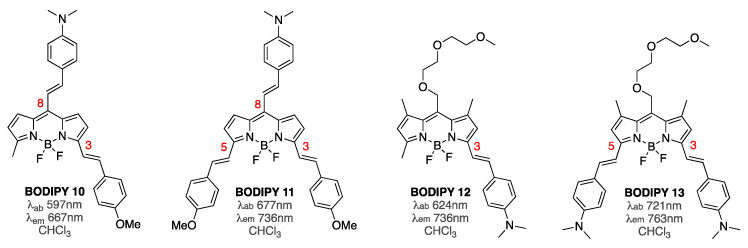
Structures of **BODIPY 10–13**.

Subsequent studies focused on exploring the BODIPY probes that featured extended conjugation not only in position 3, and both position 3 and 5, but also in the *meso* position of the BODIPY core (Figure 4) [63]. Only a 2–3-fold enhancement of the emission intensity in the presence of Aβ aggregates was noted in the presence of equimolar amounts of BODIPY sensors. However, due to the extended conjugation, the emission maxima of these dyes were shown to be in the 650–760 nm range. Considering that background interference in this spectroscopic window is typically not appreciable, smaller fluorescence enhancements by these probes might still be useful. In addition, it appeared that the position of dimethylamino-phenethyl substituent on the BODIPY core had a significant impact on both photophysical properties and affinity towards Aβ1-42 aggregates (Figure 4). Furthermore, it was demonstrated that K_d_ values did not correlate directly with the staining ability of these dyes [63]. For example, **BODIPY 12** and **BODIPY 13** exhibited somewhat close K_d_ values (i.e., around 50 nM and 100 nM, respectively), but drastically different staining capabilities. On the other hand, **BODIPY 10** and **BODIPY 12** had somewhat different K_d_ values (i.e., around 230 nM and 50 nM, respectively), yet virtually the same staining capacity. However, more detailed structure–activity relationship studies, with a broader scope of BODIPY sensors, might be required to develop general correlations between photophysical properties, staining capabilities, and binding affinities.

### 3.2. Non-Covalent Interactions of BODIPY Dyes with Aβ Oligomers

Soluble Aβ oligomers are considered the most neurotoxic forms of amyloid-β aggregates, having detrimental effects on various neuronal processes [23,24,25,26,27]. Due to the transient nature of these species, development of BODIPY probes and sensors for soluble Aβ oligomers is challenging.

**Figure 5:**
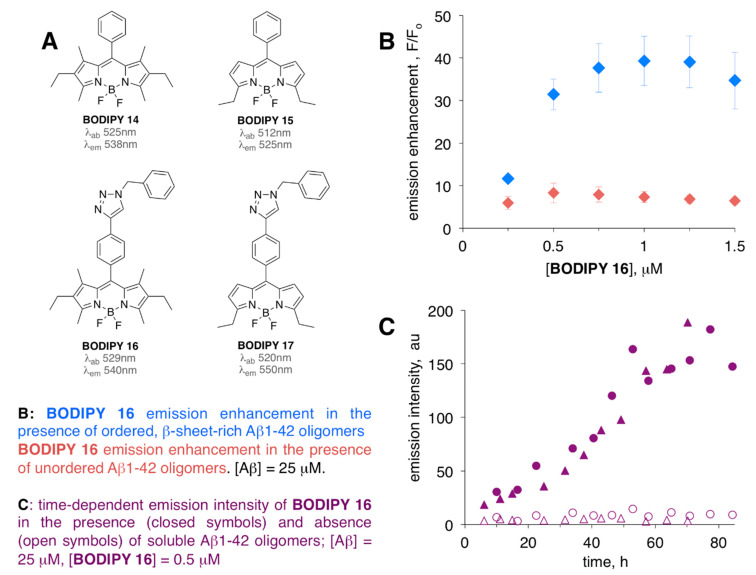
Structures of **BODIPY 14**–**17** (**A**), emission enhancement (**B**), and the time-dependent response (**C**) of **BODIPY 16** in the presence of unordered and ordered soluble amyloid-β peptide (Aβ)1-42 oligomers. Media: Tris buffer (10 mM, pH 8.7).

Some earlier studies indicated that unmodified BODIPY dyes might not be viable sensors for soluble Aβ oligomers, as relatively small (<2–3-fold) emission enhancements for **BODIPY 14** and **BODIPY 15** (Figure 5A) were noted in the present of soluble Aβ1-42 oligomers [64]. However, the introduction of a triazole-moiety in the *meso* position yielded so-called click-BODIPY dyes and led to appreciable levels (around 10-fold for unordered oligomers, around 40-fold for ordered oligomers) of fluorescent enhancement in the presence soluble oligomeric Aβ1-42 species (Figure 5B). Thus, **BODIPY 16** and **BODIPY 17** were able to detect a conformational change from unordered to ordered, yet still soluble, Aβ1-42 oligomers [64]. Time-dependent aggregation of Aβ1-42 oligomers monitored by these dyes revealed their ability to sense pre-fibrillar soluble aggregates as a continuous increase (without a lag-phase that is typically noted for ThT [65]) of fluorescent intensity was noted (Figure 5C). The dye-binding studies were in agreement with circular dichroism (CD) and light-scattering data, which were used to monitor conformational changes of Aβ1-42 oligomers from unordered, less aggregated to ordered, more aggregated, and β-sheet rich form of Aβ1-42 [66]. Importantly, (a) appreciable levels of emission intensity were achieved in the presence of large excess of Aβ1-42 as compared to the amount of **BODIPY 16** and **BODIPY 17**, and (b) neither of the dyes altered the kinetics of the Aβ aggregation process.

Thus, **BODIPY 16** was recently used in a dye-binding assay to evaluate the effect of several peptide inhibitors of Aβ1-42 oligomerization and fibrillization [67]. Significantly, **BODIPY 16** assay was contrasted with ThT-based assay [66,67], thus highlighting a complementary nature of both dyes and demonstrating the need for availability of multi-sensor approaches when addressing complex amyloid-related processes.

ThT, being a fibril-specific sensor [66,67] (as evidenced by the lag phase, i.e., no significant changes of ThT’s fluorescence intensity was observed over initial 5–6 h) [67], could not recognize oligomeric Aβ1-42 species, which were present at the early stages of Aβ aggregation, and as such ThT might not be suitable for evaluation of inhibitors of early stages of amyloid aggregation/oligomerization. On the contrary, and consistently with a previous account [64], continuous increase of **BODIPY 16** intensity was noted over 15 h of aggregation of Aβ oligomers, thus suggesting that this sensor could detect early stage Aβ aggregates [67]. In fact, using **BODIPY 16** assay, it was possible to identify a compound that could interfere with early stage, i.e., oligomeric Aβ1-42 aggregates, which, however, was inefficient in suppressing formation of Aβ1-42 fibrillar aggregates (which was assessed by using a complementary ThT-based assay). This example further illustrates the need for readily accessible, multi-targeting dye-binding assays for screening the inhibitors of various Aβ aggregation processes.

**Figure 6:**
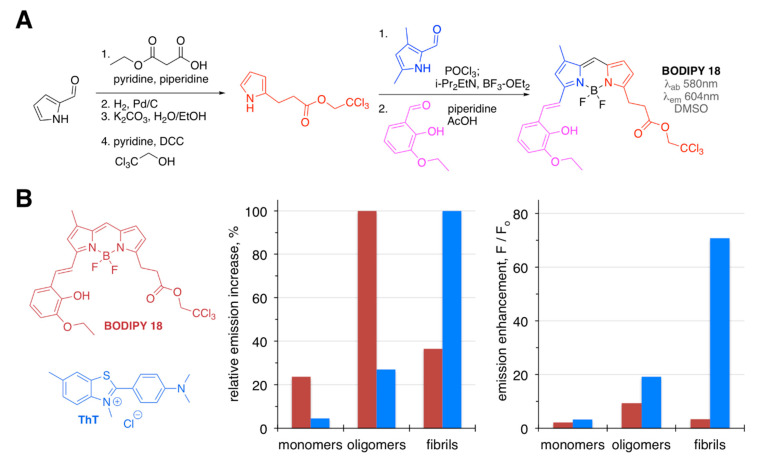
Synthesis of **BODIPY 18** (**A**), and emission intensities of **BODIPY 18** and ThT in the presence of various forms of Aβ1-42 (**B**) selectivity (left) and emission enhancement (right) towards particular forms of Aβ.

**BODIPY 18** was introduced as an Aβ1-40 oligomer-specific probe (Figure 6) [68]. This dye was prepared in several moderate-to-high yield synthetic steps [68,69,70], and it was selected using a diversity-oriented fluorescent library approach [71]. The specificity of this sensor towards oligomeric form of Aβ1-40 was around 2–3-fold higher than that towards monomeric and fibrillar Aβ1-40 species (Figure 6). On the contrary, ThT exhibited a strong preference toward fibrillar Aβ-40, with drastically higher levels of emission enhancements, when compared to **BODIPY 18** (Figure 6). Even though only a ninefold increase of the fluorescence intensity and a relatively low affinity (K_d_ = 480 nM) towards oligomeric Aβ1-40 was observed, **BODIPY 18** was proven to be a viable in vivo probe, capable of crossing the brain–blood barrier and successfully staining Aβ deposits in the transgenic mouse brain.

**Figure 7:**
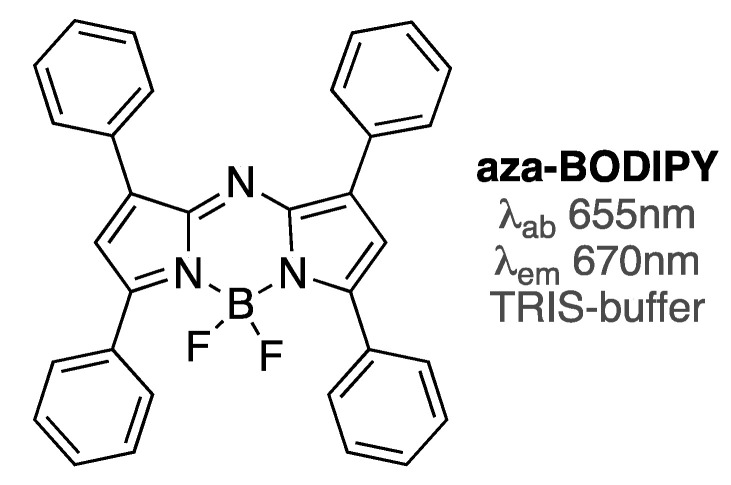
Structure of **aza-BODIPY** dye; Tris buffer (10 mM, pH 8.6).

**Aza-BODIPY** dye (Figure 7) is a viable near-IR analogue of BODIPY dyes with a broad range of applications [72]. This dye was shown to differentiate between unordered and ordered conformations of Aβ1-42 oligomers, as around 2.5-fold difference in emission intensity (with emission enhancements of sixfold for unordered oligomers, and around 16-fold for ordered oligomers) was noted [73]. Similar to click-BODIPY dyes (Figure 5), only small amounts of **aza-BODIPY** were required to attain significant emission enhancement in the presence of Aβ1-42 oligomers. Considering that these types of dyes could be synthesized in a relatively facile and straightforward manner [72,73], **aza-BODIPY** and its analogues should be viable complements to BODIPY dyes for amyloid sensing.

### 3.3. Photoinduced Electron Transfer-Based BODIPY Sensors

Ability to modulate fluorescent processes, including photoinduced electron transfer (PET) and fluorescence resonance energy transfer (FRET), by adjusting the structure of the fluorophores is of the outmost importance in designing and developing efficient fluorescence-based sensors. A number of BODIPY probes, which operate under PET mechanism that allows for molecular and ionic sensing, have been reported [74,75,76].

PET-based **BODIPY 19** was reported to sense Aβ fibrillar aggregates [77]. Specifically, a gradual decrease (over 5.5-fold at the highest concentration of Aβ) of the fluorescent intensity was observed when **BODIPY 19** (2 µM) was treated with increasing amounts of Aβ1-42 (from 0 to 2.9 µM). Notably, a similar quenching behavior was observed when **BODIPY 19** was treated with increasing amounts of Cu(II). Although the specific details on the emission-quenching mechanism remain to be clarified, it was suggested that the quenching mechanism was related to PET and FRET processes. Considering that the photophysical properties (along with metal-binding capability and biological activities) of the isomeric **BODIPY 20** were drastically different from those of **BODIPY 19** [78,79], it could be argued that conformational changes, i.e., shape, distance between BODIPY, and chelating group, might be occurring upon interaction with amyloid aggregates, thus triggering the PET-quenching mechanism.

**Figure 8:**
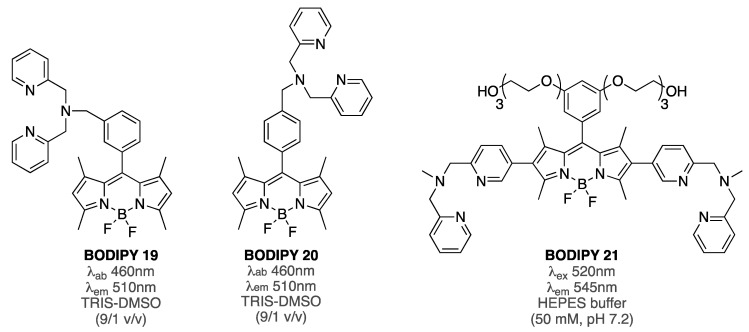
Structures of **BODIPY 19**–**21** (**BODIPY 20** had not been tested as an amyloid sensor).

The role of dipicolyl moiety in the interaction with Aβ has yet to be fully investigated, and only **BODIPY 21** (Figure 8) has shown some affinity towards Aβ aggregates (Zn^2+^-**BODIPY 21** complex exhibited EC_50_ = 650 nM, and around fourfold increase of emission intensity in the presence of Aβ fibrils, albeit without any significant staining ability) [80]. It should be noted, however, that **BODIPY 21** was designed as a tau-protein-specific probe.

Thus, it is plausible that quenching of **BODIPY 19** fluorescence, upon interaction with Aβ1-42 fibrils, was due to change of the distance and/or orientation between the metal-chelating group and the BODIPY core, rather than due to specific interactions of the amyloids with the metal-chelating group.

**Figure 9:**
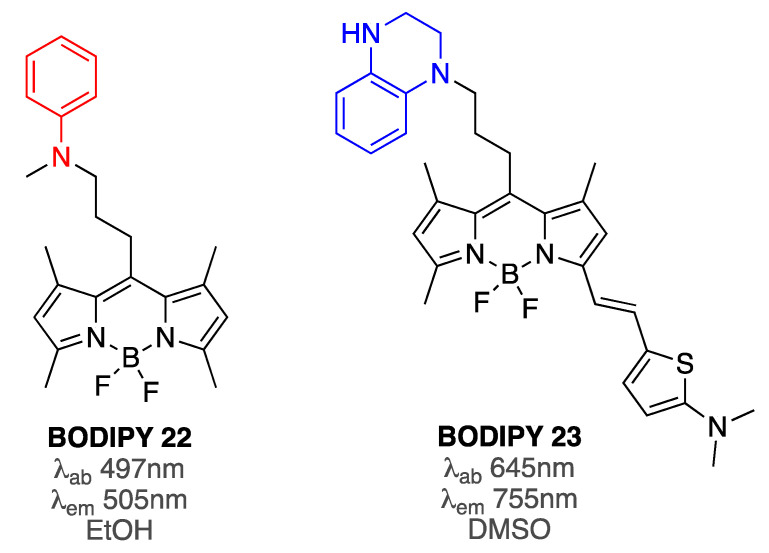
Structures of photoinduced electron transfer (PET)-based **BODIPY 22** and **BODIPY 23** sensors.

In support of the aforementioned assumption, the emission intensity of aniline-containing **BODIPY 22** upon interaction with Aβ aggregates was suggested to depend on the distance between the aniline and BODIPY group [81]. Specifically, in the free form, **BODIPY 22** was in a more compact conformation (that minimized the interactions of the hydrophobic BODIPY with the aqueous, hydrophilic media), which led to the quenching of the emission by the electron pair of the amine. On the other hand, binding to Aβ aggregates induced a more elongated conformation, which reduced or prevented PET quenching and led to increased fluorescence as a function of increasing concentration of Aβ (Figure 9). This concept was further supported by an accompanying model study in which gradual increase of steric constrains around aniline moieties, which were covalently tethered to BODIPY core, led to gradual increase of the emission intensity [81].

Subsequently, replacing an aniline group with tetrahydoquinoxaline moiety, along with incorporation of conjugation in position 3, resulted in a superior PET-based **BODIPY 23** sensor (Figure 9) [82]. Importantly, the interactions with monomeric, oligomeric, and fibrillar Aβ species were investigated. In the absence of Aβ, **BODIPY 23** was shown to be non-emissive in aqueous media. However, upon addition of increasing amounts of Aβ1-42 species, substantial increase of the emission intensity was noted, albeit with a relatively small differentiation among various forms of Aβ1-42: the fluorescence increase was 18-fold for monomers, 26-fold for oligomers, and 21-fold for fibrils. It should be pointed out that binding affinities of **BODIPY 23** towards various Aβ1-42 species were distinct, showing some preference for oligomeric Aβ1-42 (18 nM towards monomers, 6 nM toward oligomers, and 27 nM for fibrils). Importantly, during staining of the brain slices from AD mice, the staining ability of **BODIPY 23** was similar to staining ability of ab2454 antibody that could recognize various forms of Aβ1-42 [82].

### 3.4. BODIPY–Amyloid Interactions in Membrane Environments

Alteration of structural integrity of biomembranes by amyloids has been the subject of numerous studies, which established a complex nature of interactions between amyloids and membranes and their components [83,84]. Thus, studies on the fibrillization and oligomerization processes of amyloid-β in the membrane environments should aid in the further understanding of amyloid-related toxicities.

**Figure 10:**
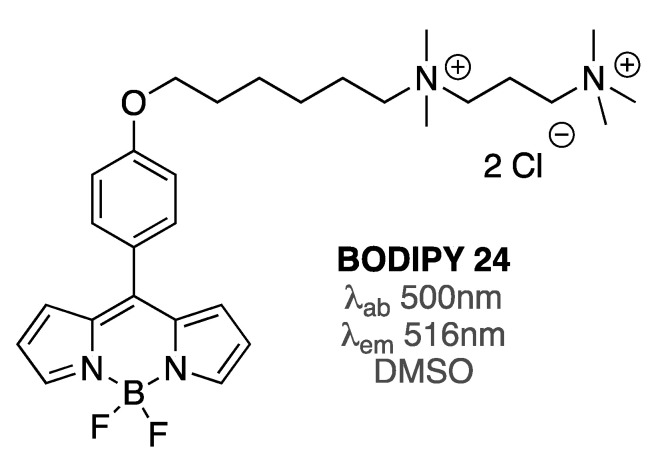
Structure of **BODIPY 24**.

Viscosity-sensitive **BODIPY 24** (Figure 10), which has been widely used as a probe to measure viscosity in membrane environments [85], was utilized to assess the effect of Aβ oligomers and fibrils on the Aβ-induced viscosity changes in several types of biomembranes, on the basis of the changes of **BODIPY 24** lifetimes [86]. Specifically, giant plasma membrane vesicles as well as live cells, such as HeLa cells and SH-SY5Y, which are generally used as model systems for neurodegenerative diseases, were utilized. In the presence of Aβ oligomers, after a few hours, membrane viscosities of giant vesicles, HeLa cells, and SH-SY5Y cells were reduced by around 30, 15, and 15%, respectively. Notably, such changes in viscosity did not result in any visible changes in the morphology of the vesicles and cells. On the contrary, Aβ fibrils did not alter the membrane viscosity of HeLa and SH-SY5Y cells, as no appreciable changes in **BODIPY 24** fluorescence lifetimes were noted [86].

Significantly, by using this BODIPY probe, it was shown that a neuroprotecting peptide could suppress the ability of Aβ1-42 oligomers to alter membranes’ viscosity of SH-SY5Y cells [86]. This result indicated that, similar to using dye-biding assays to screen for small molecule inhibitors of Aβ aggregation, utilization of BODIPY-based rotors, i.e., viscometers, could provide an additional parameter for assessing the neuroprotecting effects of various molecules directly in biologically relevant environments.

### 3.5. Covalent BODIPY-Aβ Probes

**Figure 11:**
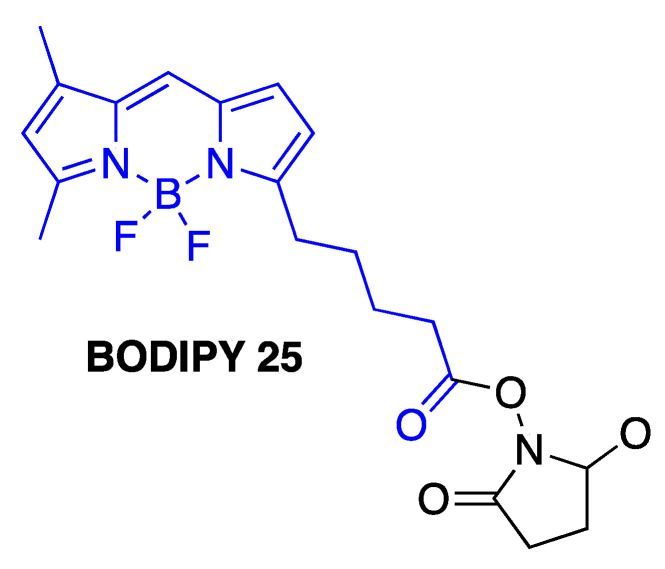
Structure of **BODIPY 25**; the portion shown in blue is conjugated to Aβ1-42.

Fluorescently-labeled Aβ1-40 and Aβ1-42 peptides have been widely used to study amyloid self-assembly processes as well as to investigate the amyloid β-related neurotoxicities [87,88,89,90,91,92,93,94]. It should be noted, however, that some recent studies indicated that the nature of fluorescent groups that are covalently linked to Aβ peptides might have a profound effect on the aggregation profile of Aβ [95,96]. Importantly, size of the fluorescent labels might not be the major factor that is contributing to the altered aggregation kinetics of Aβ species. Notably, Aβ1-42, which was labeled with **BODIPY 25** (Figure 11), was found to be the most prone to aggregation [95]. Thus, these studies highlighted the fact that even reasonable assumptions and expectations with regard to the impact of the probe on the properties of labeled amyloids (such as incorporation of uncharged amino acids in the unstructured regions of Aβ for dye conjugation, for example) should be verified using not only imaging, but also spectroscopic techniques, e.g., fluorescence correlation spectroscopy [95], which is typically used to assess various parameters of biomacromolecular interactions and protein aggregation processes [97].

**Figure 12:**
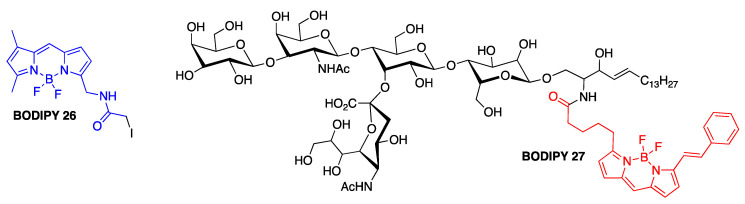
Structures of **BODIPY 26** (the portion shown in blue is conjugated to Aβ1-40) and **BODIPY 27** (shown in red).

In view of the importance of Aβ–ganglioside interactions for AD [98,99], one earlier study focused on investigating the role of various membrane components on the aggregation of mutants of Aβ1-40 [100]. Thiol-specific **BODIPY 26** was introduced on the cysteine-containing (in positions 2, 12, and 26) Aβ1-40 peptides, and together with BODIPY-labeled gangliosides (**BODIPY 27** shown as a representative example), lipids, and fatty acids, the behavior of amyloid-β in membrane- and micelle-like environments were investigated using various fluorescence methods, e.g., emission, steady-state fluorescent anisotropy and fluorescent lifetime measurements (Figure 12). It was demonstrated that gangliosides could be controlling the modes of Aβ–membrane and Aβ–micelle interactions [100].

**Figure 13:**
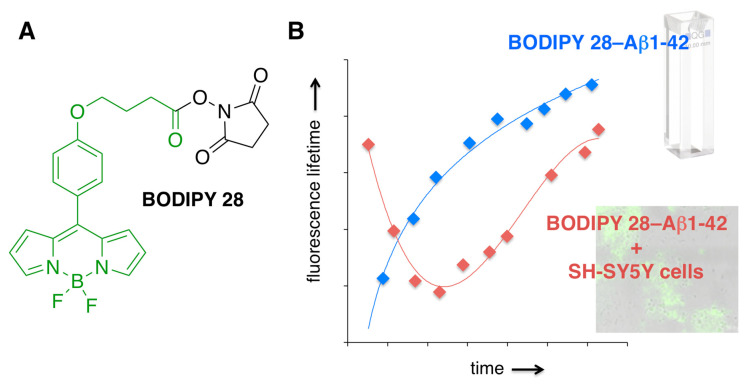
(**A**) Structure of **BODIPY 28** (only structure in green is covalently attached to an N-containing residue of Aβ1-42). (**B**) A schematic representation of time-dependent fluorescence lifetime changes of **BODIPY 28**–Aβ1-42 in the absence and presence of SH-SY5Y cell; the thin lines are only to guide the eye through the data points.

Recently, in order to assess microviscosity fluctuations upon amyloid aggregation, **BODIPY 28** was conjugated with Aβ1-42 [101] (Figure 13). This covalent attachment assured that this rotor—with these fluorescence lifetimes being sensitive to viscosity fluctuations within the environment—could report on the viscosity changes that are taking place in a close proximity of Aβ, thus providing valuable information about specific Aβ interactions with cellular components. Conjugation was conducted using naturally present lysine residues as well as the N-terminus of Aβ1-42 to produce single labeled **BODIPY 28**–Aβ1-42 construct. It should be pointed out that some of the free BODIPY could not be removed from the **BODIPY 28**–Aβ1-42 conjugate [101]. Even though the aggregation profiles of Aβ1-42 and **BODIPY 28**–Aβ1-42 appeared to be the same, as judged by tyrosine quenching assays and time-dependent TEM imaging, it is also possible that incorporation of BODIPY dye could be inducing some aggregation of Aβ, which leads to the entrapment of **BODIPY 28**.

Oligomerization of the **BODIPY 28**–Aβ1−42 construct was monitored following the changes in the fluorescence lifetime of **BODIPY 28** (Figure 13). Significantly, time-dependent microviscosity changes upon self-assembly of Aβ1-42 and/or interactions of Aβ1-42 with cellular components were shown to be drastically different from those observed upon oligomerization (aggregation) of **BODIPY 28**–Aβ1-42 in vitro [101]. Although the specific details of the Aβ self-assembly in cellulo remain to be clarified, the results unambiguously illustrated both complex nature of amyloid interactions and the need for better model systems for in vitro, i.e., in spectroscopic cell (in cuvetto) types of studies.

## 4. Conclusions

Immense structural and functional complexity of amyloid interactions requires access to probes and sensors, which possess tunable, diverse characteristics. Arguably, virtually unsurpassed versatility of BODIPY dyes, with regard to synthesis and tunable photophysical properties, makes them one of the privileged scaffolds for the development of probes and sensors for amyloids. Available literature accounts have already firmly established the ability of these dyes to sense various fibrillar and oligomeric forms of Aβ peptides (Table 1), not only in relatively simple model buffer conditions, but also in in vitro; in cellulo; ex vivo; and, most importantly, in vivo settings.

These studies should provide fruitful foundation for further improvements of sensing ability of BODIPY dyes, not only towards Aβ peptides, but also other types of amyloids. Considering ever-improving synthetic methods allowing for introduction of ever-expanding functionalities, it is highly likely that BODIPY dyes could be the torch that lights the path to solve the challenging problems of amyloids’ behavior in various types of environments.

## Figures and Tables

**Table 1:** A list of BODIPY sensors used to date to detect Aβ peptide species.

Aβ	BODIPY	Reference
Fibrils/aggregates/AD homogenates		
	BODIPY 4	[60]
	BODIPY 5	[61]
	BODIPY 6–9	[62]
	BODIPY 10–13	[63]
	BODIPY 19	[77]
	BODIPY 21	[80]
	BODIPY 22	[81]
	BODIPY 24	[85]
		
Oligomers		
	BODIPY 16	[64,67]
	BODIPY 18	[68]
	BODIPY 23	[82]
	BODIPY 24	[85]
